# Longitudinally Profiling Neutralizing Antibody Response to SARS Coronavirus with Pseudotypes

**DOI:** 10.3201/eid1103.040906

**Published:** 2005-03

**Authors:** Nigel J. Temperton, Paul K. Chan, Graham Simmons, Maria C. Zambon, Richard S. Tedder, Yasuhiro Takeuchi, Robin A. Weiss

**Affiliations:** * University College London, London, United Kingdom; † Prince of Wales Hospital, Shatin, New Territories, Hong Kong Special Administrative Region, People’s Republic of China; ‡ University of Pennsylvania School of Medicine, Philadelphia, Pennsylvania, USA; § Health Protection Agency Central Public Health Laboratory, London, United Kingdom

**Keywords:** SARS virus, Neutralization tests, Infectious diseases, emerging, Vaccines, research

## Abstract

The severe acute respiratory syndrome–associated coronavirus (SARS-CoV) spike protein (S) is a major target for neutralizing antibodies. Retroviral SARS-CoV S pseudotypes have been constructed and used to develop an in vitro microneutralization assay that is both sensitive and specific for SARS-CoV neutralizing antibodies. Neutralization titers measured by this assay are highly correlated to those measured by an assay using replication-competent SARS-CoV. No cross-neutralization occurred with human sera known to contain antibodies to coronavirus strains OC43 and 229E. The pseudotype assay was used to profile neutralizing antibody responses against SARS-CoV S in sequential serum samples taken from 41 confirmed SARS patients during the 2003 outbreak in Hong Kong and shows long-lasting immunity in most recovered patients. The pseudotype assay does not require handling live SARS virus; it is a useful tool to determine neutralizing titers during natural infection and the preclinical evaluation of candidate vaccines.

## Abstract 2

SARS-CoV spike protein pseudotypes are the basis of an in vitro microneutralization assay sensitive and specific for SARS-CoV neutralizing antibodies.

The coronavirus that causes severe acute respiratory syndrome (SARS-CoV) is a new human pathogen for which a vaccine may be urgently required should a new outbreak occur. Studying the magnitude and longevity of the neutralizing antibody response during natural infection will help establish correlates of protection to be generated by immunization. Humoral immunoglobulin (Ig) G, IgM, and IgA responses to SARS-CoV have been studied extensively (*1*–*7*). However, studies of neutralizing antibody responses during natural infection have been limited (*8*,*9*), partially because neutralization assays must be performed at biosafety level 3 or higher.

The SARS-CoV genome encodes 4 structural proteins, the spike (S), membrane (M), envelope (E), and nucleocapsid (N) proteins (*10*). The S protein is the major surface antigen of the virus, and the neutralizing antibody response is primarily directed against this protein. Monoclonal antibodies to the S protein neutralize the virus and have been mapped (*11*–*14*). By vaccinating hamsters with a recombinant parainfluenza virus vector, Buchholz et al. found that the expression of M, E, or N, in the absence of S, did not induce a neutralizing antibody response (*15*). Preclinical studies of SARS-CoV vaccines provide evidence that generating a strong neutralizing antibody response to SARS-CoV S may protect against SARS infection (*16*–*19*).

Retroviral and lentiviral pseudotypes have been employed in lieu of replication-competent virus to study neutralizing antibody responses to viral infection (*20*,*21*). Pseudotype viruses encode marker genes and bear foreign viral envelopes (*22*). The transfer of marker genes to target cells depends on the function of the E protein; therefore, the titer of neutralizing antibodies against the envelope can be measured by a reduction in marker genes transferred. Lentiviral pseudotypes bearing the SARS-CoV spike protein were first described by Simmons et al. to study viral entry (*23*). Other studies have used SARS-CoV S pseudotyped viruses for identifying receptors (*24*), examining viral tropism (*25*–*27*), and measuring neutralizing antibody responses (*18*,*28*–*30*). Yang et al. constructed lentiviral pseudotypes harboring S, M, or E proteins and found that only S supported viral entry into target cells (*26*).

The aim of this study was to establish a neutralizing antibody assay using murine leukemia virus (MLV) pseudotypes bearing the SARS-CoV S envelope, MLV(SARS), and to profile neutralizing antibody responses to SARS-CoV natural infection during a relatively long period in a cohort of Hong Kong patients who had recovered from the disease.

## Materials and Methods

### Patient Samples

A total of 166 blood samples were obtained from 41 patients (68% female) 11–80 years of age who were admitted to the Prince of Wales Hospital, Hong Kong, from March to May 2003. All study patients fulfilled the World Health Organization criteria for having a probable case of SARS. Samples from 7 of the 41 patients were tested for SARS-CoV by reverse transcription–polymerase chain reaction (RT-PCR) in a study previously described (*31*), and 4 patients had positive results. Pneumonia developed in all 41 patients, and 6 required intensive care. None of these patients died of the infection. For most patients, multiple samples were obtained at sequential times covering the acute, convalescent, and recovered phase of the disease. This study was approved by the Prince of Wales Hospital local institutional ethics committee.

### Plasmids and Cell Lines

Construction of the plasmid pCAGGS-S harboring full-length SARS-CoV S from the Urbani strain has been described previously (*23*). The MLV gag/pol construct, pCMVi, and the green fluorescent protein (GFP) reporter construct, pCNCG, have been described (*32*). Vesicular stomatitis virus E protein (VSV-G) expression vector pMDG has been described previously (*33*). HIV constructs were used as described (*34*).

All cell lines were cultured in Dulbecco’s Modified Eagle Medium (DMEM) with Glutamax and high glucose (Gibco, Paisley, Scotland, UK), supplemented with 10% fetal calf serum and penicillin/streptomycin. To make the quail QT6/ACE2 cell line, the gene encoding the receptor for SARS-CoV, human angiotensin-converting enzyme 2 (ACE2) (*35*), was cloned from a human primary kidney cDNA library (Invitrogen, Paisley, Scotland, UK) using 21-mer primers designed to the start and stop of ACE2, and subcloned into pcdna3.1+. QT6 cells were transfected by using lipofectamine 2000 and selected with G418, and a bulk ACE2-positive, G418-resistant population was grown.

### Viral Vector Production and Infection of Target Cells

Confluent plates of 293T cells were split 1:4 the day before transfection. Each plate of 293T cells was transfected with 1 μg gag/pol construct, 1.5 μg of enhanced GFP reporter construct, and 1.5 μg envelope-expressing construct by using the Fugene-6 transfection reagent (*36*). Supernatant was harvested 48 h and 72 h posttransfection, filtered through 0.45-μm filters, and stored at –80^o^C. MLV and HIV vector titer were measured on 293T, TE671, and QT6/ACE2 cells and are presented as infectious units (IU) per milliliter. Briefly, cells were infected with vector, and eGFP titers were determined 72 h later by fluorescence-activated cell sorter (FACS).

### Neutralization Assays

#### Live Virus

Patient serum samples were heat inactivated at 56°C for 30 min and serially diluted from 1:10 in culture medium. Fifty PFU of SARS Frankfurt strain were added to the serum dilution and incubated for 1 h at 37°C. We added 5 × 10^4^ Vero E6 cells per well to the virus and serum mix, and the mixture was incubated in 96-well plates for 4 days, after which neutralization was assessed by cytopathic effect (CPE). The neutralization endpoint was taken as the last well in which complete neutralization was observed. Serum samples were assayed in duplicate, and positive results were confirmed in separate assays.

### Pseudotype

Patient serum samples were heat inactivated at 56°C for 30 min, 2-fold serially diluted from 1:10 in culture medium, and mixed with MLV(SARS) virions (≈100 IU) at a 1:1 vol/vol ratio. After incubation at 37°C for 1 h, 100 μL of each dilution was added to QT6/ACE2 cells seeded at 1 × 10^4^ cells per well in 96-well flat-bottomed tissue culture plates seeded 24 h previously. GFP-positive cells were counted 48 h later by fluorescence microscopy. Neutralizing antibody titers are presented as geometric mean titers of assays performed in triplicate.

## Results

### Production of MLV S Pseudotypes

**Figure 1:**
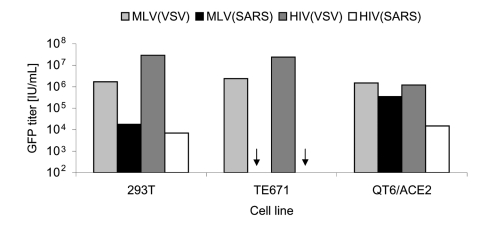
Infectivity of retroviral severe acute respiratory syndrome–associated coronavirus (SARS-CoV) spike protein (S) pseudotypes on target cells. SARS-CoV S-mediated infection of human 293T, TE671, and Quail QT6/ACE2 was assessed. Murine leukemia virus (MLV) or HIV pseudotypes bearing either the pantropic vesicular stomatitis virus envelope protein (VSV-G) as a positive control, or the SARS-CoV S, were added to target cells. After 72 h, green fluorescent protein (GFP)–positive cells were counted by fluorescence-activated cell sorter analysis. Infection titers are given as infectious units per milliliter (IU/mL). Arrow indicates that infection titer was less than the detection limit, 10^2^ IU/mL.

Retroviral particles pseudotyped with SARS-CoV S were made by cotransfection of an S-expressing plasmid, pCAGGS-S, with plasmids encoding MLV or HIV gag-pol and GFP vector genome in 293T cells. Culture supernatants were used to infect human TE671, 293T, and quail QT6/ACE2 cell lines. VSV-G pseudotyped MLV particles, MLV(VSV), and HIV particles, HIV(VSV), were used as controls. MLV(VSV) and HIV(VSV) pseudotypes infected all 3 cell lines tested. MLV(SARS) and HIV(SARS) pseudotypes infected 293T (which have a low level of endogenous ACE2 expression) and QT6/ACE2 but not TE671 cells (Figure 1). The highest titer (3.5 × 10^5^ IU/mL) was obtained with the combination of QT6/ACE2 cells and MLV(SARS), so this system was employed for all subsequent assays.

### Validation of Pseudotype Microneutralization Assay

**Figure 2:**
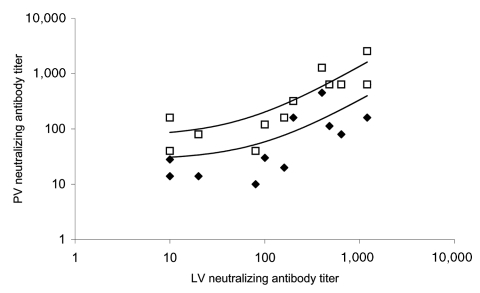
Correlation of neutralizing antibody titers measured by plaque reduction assay with titers measured with pseudotype assay. LV, neutralizing antibody titer by using replication-competent severe acute respiratory syndrome–associated coronavirus (SARS-CoV) (live virus); PV, neutralizing antibody titer by using pseudotype virus; PV_90_ (filled black diamonds), 90% neutralizing antibody titer by using murine leukemia virus (MLV) (SARS) pseudotype virus; PV_50_ (open squares), 50% neutralizing antibody titer. Logarithmic trendlines were fitted to the data by using Microsoft Excel 2003 (Microsoft Corp., Redmond, WA, USA). Correlation coefficients for LV versus PV_90_ and LV versus PV_50_ are 0.69 and 0.78, respectively.

A blinded panel of 50 samples comprising sera from healthy persons, patients infected with other human coronaviruses (OC43 and 229E), patients infected with influenza virus, and persons who were convalescent from SARS was provided by the Health Protection Agency (HPA), United Kingdom, for the validation of our pseudotype neutralization assay. For 12 samples positive for both assays, 90% and 50% inhibitory concentration (IC_90_ and IC_50_) pseudotype neutralizing titers were compared with titers obtained at HPA by neutralization assay using replication-competent SARS-CoV. Logarithmic plots of pseudotype versus live virus neutralization titers are shown in Figure 2. Correlation coefficients for pseudotype IC_90_ and IC_50_ titers versus live SARS-CoV neutralization titers were 0.69 and 0.78, respectively. MLV(SARS) entry into QT6/ACE2 cells was not substantially inhibited by sera from healthy persons or from persons with human coronavirus OC43 and 229E antibodies. MLV(VSV) infection was not inhibited by any sera (data not shown). The pseudotype assay was thus shown to be both sensitive and specific for SARS-CoV neutralizing antibodies, with no evidence for cross-reaction with the other human coronaviruses. Although the live virus assay was based on the Frankfurt SARS-CoV isolate, and the pseudotype assay was based on the Urbani isolate, they gave equivalent titers, including analysis of serum from the person from whom the Frankfurt isolate was made.

### Neutralizing Antibody Response to SARS-CoV S

**Figure 3:**
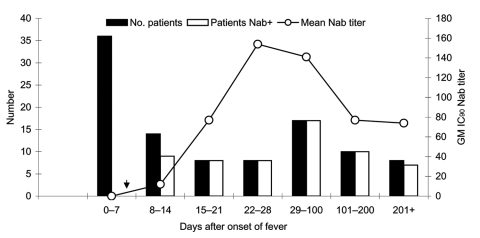
Severe acute respiratory syndrome–associated coronavirus (SARS-CoV) neutralizing antibody–positive rate by time of blood sample collection (days after onset of fever). Black bars represent the number of patients tested for neutralizing antibodies (Nab). White bars represent the number of patients whose assayed samples were positive for neutralizing antibodies (Nab+). Samples are considered positive for Nab if the 90% neutralizing antibody titer determined by using murine leukemia virus (MLV) (SARS) pseudotypes is >10. Line plot with open white circles shows the geometric mean (GM) Nab titer within each time frame. IC_90_, 90% inhibitory concentration.

**Figure 4:**
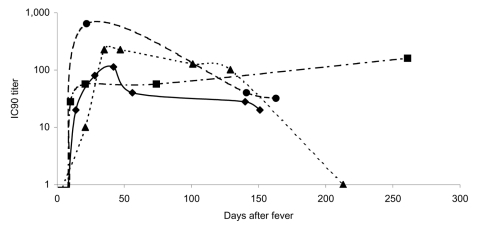
Neutralizing antibodies to severe acute respiratory syndrome–associated coronavirus spike protein in sequential blood samples from 4 representative patients. Lines represent profiles of individual patients. Filled black symbols represent geometric mean titers at individual time points. IC_90_, 90% inhibitory concentration.

Blood samples from the Hong Kong cohort of patients were tested for neutralizing antibodies to the SARS-CoV S protein by using the pseudotype neutralization assay. Figure 3 shows the number of patients positive for neutralizing antibodies and the mean neutralizing antibody titer displayed by week after onset of fever. Samples taken during the convalescent and recovered phase (after day 28 following onset of fever) are grouped into longer time blocks (29–100 days, 101–200 days, and >201 days). In the first week after onset of fever, all patient samples tested were negative for neutralizing antibody. Appearance of neutralizing antibody was first seen in week 2 with 9 (64%) of 14 patients becoming positive. Geometric mean IC_90_ neutralizing antibody titers ranged from negative (<10) to 40. In week 3, all patients were positive for neutralizing antibodies with titers from 10 to 200. IC_90_ titers peaked during week 4 (mean titers 28–640) but persisted in some patients for >200 days after onset of fever. Figure 4 shows the longitudinal profiles of neutralizing antibody responses to SARS-CoV S in 4 representative patients for whom serially collected blood samples were available for testing.

## Discussion

We have developed a retroviral pseudotype-based assay that facilitates the accurate determination of neutralizing antibody responses to SARS-CoV without the use of replication-competent virus. Since the neutralization titers measured on replication-competent SARS-CoV and pseudotypes are highly correlated, this assay can be widely applied in routine diagnostics and used for the preclinical evaluation of candidate vaccines and immune therapies for SARS, without the pathogen itself being handled. This advantage is important because nosocomial infections have arisen from laboratory handling of SARS-CoV in Taiwan, Singapore, and Beijing (*37*). A lack of good, quantitative assays for SARS-CoV replication in vitro also makes the pseudotype assay, with its easily interchangeable reporter genes, a more flexible platform with which to study neutralization and cell tropism.

Our assay detected neutralizing antibodies generated during both the acute and convalescent phases of SARS infection. When looking for neutralizing antibody responses, previous researchers have predominantly tested samples taken during the convalescent phase of the disease, whereas we found that during the period 8–14 days after onset of fever, 9 patients in our cohort had neutralizing responses to SARS S protein. Viral load, as measured by real-time RT-PCR, for 19 of the patients in our cohort, was previously shown to peak at approximately day 4 or 5 after onset of fever and then decreased to barely detectable around the time of seroconversion (*38*), which suggests that the neutralizing antibody response may play a role in viral clearance. This finding has implications for diagnostics and surveillance, since positive diagnoses for neutralizing antibodies can be made earlier in infection and as a complement to testing for IgG responses by enzyme-linked immunosorbent assay. SARS has yet to manifest itself as a seasonal epidemic threat like influenza, which makes mass vaccination an unlikely scenario. The rapid detection of neutralizing antibodies seen in this study suggests that localized vaccination with an effective vaccine is likely to help control the spread of SARS-CoV during an outbreak, if vaccine elicits as rapid a response as live virus.

This article also reports longitudinal neutralizing antibody profiles in patients with SARS by using blood samples collected at serial time points (up to day 287). A broad spectrum of longitudinal profiles is seen in patients, and neutralizing antibody levels persist in many recovered persons for several months (Figure 4). In only 1 patient did we find a complete loss of neutralizing antibody titer after a sharp rise, which began at the end of the acute phase (day 10). In a second patient, IC_90_ neutralizing antibody titers attained 640 by day 22 after onset of fever, followed by a decline; however, in another patient, neutralizing antibody was detectable at day 261 after onset of fever. Maintenance of neutralizing antibody titers will have important implications for vaccine design.

Gao et al. (*39*) tested in rhesus macaques an adenoviral vaccine that was made up of the S1 spike fragment, M, and N; the test showed that strong neutralizing antibody responses were generated, some of which appeared early after vaccination. We have shown that some patients convalescing from SARS have similar responses before full recovery, which suggests that this level of vaccine-induced neutralizing antibodies may be protective. Initial preclinical studies in mice and hamsters are encouraging and show that neutralizing antibodies are sufficient to protect against live virus challenge (*16*–*19*,*40*). Candidate vaccines for SARS must be moved from the preclinical evaluation phase to clinical trials in human volunteers as rapidly as possible, since the possibility of further SARS outbreaks is uncertain. The method used here to analyze natural infection can be applied to clinical trials of candidate vaccines, and we expect this test to be equally applicable to animal sera.
